# The HIV-1 capsid and reverse transcription

**DOI:** 10.1186/s12977-021-00566-0

**Published:** 2021-09-25

**Authors:** Christopher Aiken, Itay Rousso

**Affiliations:** 1 Department of Pathology, Microbiology and Immunology, Vanderbilt University Medical Center, Nashville, TN USA; 2 Department of Physiology and Cell Biology, Ben-Gurion University of the Negev, Beer Sheva, Israel

**Keywords:** HIV-1, Capsid, Reverse transcription, IP6, Stiffness, Uncoating

## Abstract

The viral capsid plays a key role in HIV-1 reverse transcription. Recent studies have demonstrated that the small molecule IP6 dramatically enhances reverse transcription in vitro by stabilizing the viral capsid. Reverse transcription results in marked changes in the biophysical properties of the capsid, ultimately resulting in its breakage and disassembly. Here we review the research leading to these advances and describe hypotheses for capsid-dependent HIV-1 reverse transcription and a model for reverse transcription-primed HIV-1 uncoating.

## Background

During infection by retroviruses, including the human immunodeficiency virus type 1 (HIV-1), the positive-sense viral RNA genome is copied into double-strand DNA by reverse transcription. This reaction is catalyzed by the viral reverse transcriptase enzyme (RT), which synthesizes both the negative and positive-sense DNA strands and generates the DNA sequence elements required for integration and subsequent expression of the integrated provirus. Reverse transcription is an essential step in HIV-1 infection, a major antiviral target, and a biological process of great interest. Research during the past 20 years has revealed that the viral capsid plays an essential role in reverse transcription, yet the mechanism for this is unknown. Moreover, reverse transcription appears to induce structural changes in the capsid. Here we describe the research toward an understanding of the role of the capsid in reverse transcription and highlight recent advances in this field.

## Organization of the HIV-1 core

**Fig. 1:**
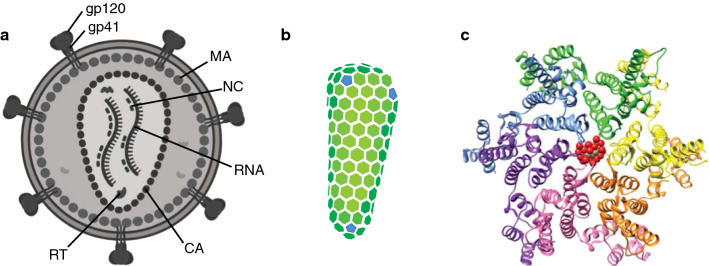
Organization and structure of the HIV-1 capsid. **A** Schematic of a mature HIV-1 particle in cross-section, with protein layers labeled (generated from BioRender). **B** Geometric model for the arrangement of CA subunits in the mature viral capsid. The CA hexamers are colored green and pentamers blue. Diagram adapted from [16]. **C** Structure of the CA hexamer with bound IP6, rendered from pdb 6BHT [4]. Phosphate oxygens in IP6 are shown as red spheres

Human immunodeficiency virus type 1 (HIV-1) particles contain a nucleoprotein core surrounded by a lipid membrane (Fig. 1A). The core consists of two copies of viral genomic RNA in association with other virion components including the nucleocapsid (NC), integrase (IN), and reverse transcriptase (RT) proteins. This ribonucleoprotein complex is encased within the viral capsid. The capsid is an elegantly simple structure consisting of the capsid protein (CA) assembled into a lattice of ~ 200 hexamers and exactly 12 pentamers organized according to fullerene cone geometry (Fig. 1B). Asymmetric placement of the pentamers gives rise to the conical capsid shape that is characteristic of members of the *lentiviridae* genus of retroviruses to which HIV-1 belongs. Although the capsid morphology is different in other retrovirus genera, the basic geometric principles of capsid structure (lattice of hexamers with 12 pentamers) is conserved among retroviruses. The particular distribution of the pentamers is what determines the shape of the particular viral capsid.

## The capsid results from maturation of the core

The HIV-1 capsid is formed during particle maturation, a late step in replication that is initiated during particle budding from infected cells and completed soon thereafter (reviewed in [1]). Maturation requires accurate proteolytic cleavage of the polyproteins Gag and Gag-Pol from which the virion is assembled. Cleavage produces the individual viral proteins MA, CA, NC, and p6 as well as RT and IN. The individual polyprotein cleavages occur at different rates, giving rise to the specific structures within the virion. CA is liberated the most slowly, suggesting that the other Gag cleavage products first form a complex with the viral RNA prior to closure of the capsid which depends on release of the C-terminal spacer peptide (SP1) from CA [2]. Interestingly, recent studies have shown that binding of IN to the viral RNA is critical for proper targeting of the viral RNA to the interior of the HIV-1 core, suggesting that this interaction plays a key role of the core morphogenesis [3]. During HIV-1 particle formation, the cellular metabolite inositol hexakisphosphate (IP6) binds to the assembling (i.e., immature) Gag lattice, stabilizing it and leading to IP6 incorporation within virus particles [4, 5]. In cells depleted of IP6 and its precursor inositol pentakisphosphate (IP5), HIV-1 assembly is impaired [6, 7]. During maturation, IP6 is thought to dissociate from the disassembling immature lattice and to subsequently promote capsid assembly by coordinating a conserved arginine side chain in the center of the CA hexamer as the mature capsid is formed [4] (Fig. 1C).

## Capsid stability and HIV-1 infection

HIV-1 infection is initiated by fusion of the viral and cell membranes, resulting in release of the viral core into the cytoplasm. The important role of the viral capsid in HIV-1 reverse transcription was first noted some 20 years ago, but only recently has its mechanism become amenable to biochemical analysis. Initial structural studies of the capsid were hampered by challenges associated with crystallization of the full-length CA protein likely owing to its two-domain flexible structure [8, 9]. A key breakthrough in structural analysis of the HIV-1 capsid was the observation that purified CA can self-assemble into tubular structures consisting of hexameric lattices [10, 11]. Analysis of these assemblies by cryoelectron microscopy revealed the subunit interfaces [12], and individual structures of CA pentamers and hexamers were obtained by engineered CA–CA crosslinking and X-ray crystallography, ultimately yielding a structural model of the entire HIV-1 capsid [13, 14]. Advances in cryoEM technology coupled with molecular dynamics simulations ultimately yielded improved structures of the capsid [15, 16]. These studies provided informative views of the arrangement of subunits and the intersubunit interfaces that contribute to capsid stability.

Functional studies of the capsid followed a parallel track. Early work revealed that CA substitutions that result in morphologically aberrant capsids impair HIV-1 infectivity and reverse transcription [17–22]. Subsequently, Forshey et al*.* characterized a series of HIV-1 mutants containing substitutions in the CA protein in an effort to determine the role of the capsid in early steps of HIV-1 infection [23]. Cores were isolated by detergent treatment of HIV-1 particles and the levels of associated CA were quantified. Cores from HIV-1 mutants that retained substantial quantities of CA were further assayed for spontaneous uncoating in vitro. That study identified several mutants with unstable capsids, including substitutions in the amino-terminal domain (R18A/N21A, P38A, Q63/Q67A, and R143A) and several in the carboxyl-terminal domain (R143A, K170A, K203A, and Q219A), most of which also exhibited impaired reverse transcription. Surprisingly, two mutants (E45A, E128A/R132A) contained capsids with enhanced stability. By contrast, a set of five CA mutants that exhibited infectivity values similar to wild type virus had capsids of normal stability. Subsequent work identified additional substitutions that alter capsid stability, including changes in Y145 in the hinge region connecting the two CA domains [15, 24]. Hence, both decreased as well as increased capsid stability were linked to reduced HIV-1 infectivity. The hyperstable E45A mutant was initially reported as defective for reverse transcription in target cells. However, a subsequent study showed that E45A is competent for reverse transcription but impaired for nuclear entry [25]. Specific CA substitutions at the three-fold interhexamer interface were observed to stabilize the viral capsid and reduce infectivity [15]. Collectively, these studies established that the stability of the capsid must be properly balanced for infection to occur and that capsid destabilization via perturbation of intersubunit interfaces normally results in impaired reverse transcription. These conclusions were further supported by studies showing that premature HIV-1 uncoating induced by restrictive tripartite motif 5 (TRIM5) proteins or small molecule antiviral compounds is associated with impaired reverse transcription [26–30]. Collectively, these early studies demonstrated that the conical capsid structure and the metastability of the viral capsid are important for optimal HIV-1 replication.

## Potential mechanisms of capsid-dependent reverse transcription

How might the capsid promote HIV-1 reverse transcription in target cells? Although this question is not yet answered, the capsid may perform several functions in early stages of infection. (i) The capsid may serve as a molecular reaction vessel for reverse transcription. The capsid is a closed volume composed of ~ 1200 molecules of CA. By contrast, it is estimated that ~ 50 molecules of active RT are present within the virion [31] with a fraction of that residing within the core. RT is a relatively slow polymerase that dissociates from the template repeatedly during DNA synthesis in vitro [32]. The dimeric viral genomic RNA adopts a complex secondary structure [33], and reverse transcription involves two template-switching events. Thus, completion of reverse transcription likely requires repeated reassociation of RT with the nucleic acid template. Therefore, one plausible hypothesis is that a closed viral capsid serves to maintain sufficient concentrations of RT and possibly other core components within the core during the several hour process of reverse transcription. A putative container function of the capsid may also ensure efficient recombination during reverse transcription by ensuring rebinding of RT after its dissociation from a template. (ii) As a container, the capsid could also exclude deleterious cytoplasmic molecules, including nucleases that may degrade the viral nucleic acids as well as DNA-sensing proteins that might alert the cell to the presence of reverse transcribed DNA in the cytoplasm and trigger an innate antiviral response. (iii) The capsid may also serve as a vehicle to promote the intracellular transport of the viral core to its destination in the nucleus. Studies in the past several years have confirmed these latter hypotheses, demonstrating that the capsid binds a myriad of host cell proteins that contribute its functions in cloaking the viral DNA from the cytoplasmic DNA sensor cyclic GMP-AMP synthase [34, 35] and serves as a docking platform for host proteins that link the core to the microtubule-based transport machinery to facilitate intracytoplasmic transport of HIV-1 to its destination, the nucleus [36–41]. In additional support of the container hypothesis, Eschbach et al*.* recently demonstrated that HIV-1 mutants with unstable capsids undergo degradation of their viral RNA and IN in target cells, in addition to losing RT [42].

Recent biochemical studies have strengthened the conclusion that the viral capsid plays a direct role in HIV-1 reverse transcription. For decades, researchers noted that HIV-1 reverse transcription is inefficient in vitro. Endogenous reverse transcription (ERT) assays were performed by incubating virions with dNTPs in the presence of detergents to permeabilize the viral membrane. In these reactions, only a fraction of viral genomes is copied into DNA, and the reactions produce few if any full-length DNA molecules. By contrast, ERT reactions with murine and avian retroviruses can efficiently generate full-length DNA molecules capable of producing a spreading infection upon transfection of permissive host cells [43–46]. These observations prompted efforts to identify human cell factors that enhance HIV-1 ERT. The Harrich group detected an ERT-promoting activity in cell extracts that copurified with translation elongation factors 1A and 1G (eEF1A and eEF1G), abundant cellular proteins involved in translation [47, 48]. Depletion of eEF1A from target cells resulted in decreased reverse transcription, and further studies indicated that the host factors promote HIV-1 infection by binding to RT and facilitating reverse transcription in target cells [49]. Despite these interesting observations, it appears that other research teams have not investigated the role of host cell translation initiation factors in HIV-1 infection.

## IP6 as a molecular linchpin

As mentioned previously, recent attention has been focused on the small molecule IP6, an abundant eukaryotic cell metabolite. IP6 is synthesized from phosphatidylinositol (4,5)-bisphosphate in a series of reactions by host enzymes including the inositol polyphosphate multikinase (IPMK) and inositol pentakisphosphate 2-kinase (IPPK) (reviewed in [50]). IP6 plays a role in diverse cellular processes. An effect on HIV-1 particle assembly was first noted in an early study which showed that both IP6 and its precursor IP5 can promote the proper assembly of HIV-1 virus-like particles in vitro [51]. IP6 was subsequently shown to bind to a critical structural element in the Gag polyprotein (the six-helix bundle) that is disassembled upon proteolytic processing of Gag during HIV-1 maturation [4]. This led to the hypothesis that polyanions like IP6 play a role in capsid assembly and stabilization by binding to the six arginine side chains located within the center of the CA hexamer. In support of this hypothesis, it was demonstrated that IP6 binds to CA hexamers in vitro and enhances their thermal stability [4, 5]*.* Furthermore, addition of IP6 to purified recombinant CA protein resulted in a dramatic enhancement of CA assembly yielding high quantities of cone-shaped capsids resembling native HIV-1 cores [4, 5]. These findings helped explain earlier observations that mutations in CA in the central region of the assembled hexamer are deleterious to HIV-1 capsid assembly [52]. The results support a role of virion associated IP6 in capsid assembly and a possible function in target cells. Although it appears that the major role of IP6 in the HIV-1 life cycle is at the stage of virion assembly [6, 7], an effect in target cells also seems plausible given the strong effects of IP6 on the ERT reaction, as described below. One interesting possibility is that stabilization of the capsid by IP5 and IP6 modulates the effects of capsid-targeting antiviral compounds, such as PF74 and GS-CA1 [30, 53].

## IP6 promotes efficient HIV-1 ERT

Recently, Christensen and coworkers identified reaction conditions in which HIV-1 particles undergo efficient ERT [54]. The authors permeabilized mature HIV-1 virus-like particles using the natural bee venom peptide melittin, thus avoiding the potential inhibitory effects of detergents. They selected reaction conditions based on the known concentrations of intracellular metabolites including nucleotides and IP6 as well as a cell extract. These conditions resulted in robust synthesis of viral DNA with ~ 50% of templates copied into full-length molecules, and addition of IP6 was critical for this high efficiency. Analysis of a set of CA mutants exhibiting altered capsid stability revealed altered dependence on optimal IP6 concentrations in the reactions, further supporting the view that the ERT enhancement by IP6 results from capsid stabilization. In an independent study, Jennings et al. optimized ERT reactions with purified HIV-1 cores, observing that addition of physiological concentrations IP6 resulted in efficient ERT mainly by enhancing the synthesis of the complete minus strand [55]. They showed that IP6 directly stabilizes the viral capsid and reduces the dissociation of RT from HIV-1 cores. In both studies, addition of a capsid-destabilizing antiviral compounds was shown to inhibit ERT, further strengthening the conclusion that a stable capsid is needed for efficient HIV-1 reverse transcription. An important message from these studies is that the viral capsid plays a direct and essential role in HIV-1 reverse transcription and that IP6 may be important for capsid function in target cells. Christensen et al. also showed that the ERT reactions supported integration of the reverse transcribed HIV-1 DNA in vitro, yielding a new technology that may facilitate the biochemical analysis of HIV-1 integration in the natural context of the viral nucleoprotein complex.

Despite the strong requirement for IP6 observed in ERT reactions, depletion of IP6 and its precursor IP5 from target cells resulted in little to no reduction in the early steps of HIV-1 infection. Mallery and coworkers disrupted the IPMK gene in 293T cells and evaluated the consequences for HIV-1 infection [6]. Infection of the knockout cells was not substantially reduced, indicating that IPMK (which converts IP3 into IP5, the precursor to IP6) is not essential for HIV-1 reverse transcription in this cell line. Sowd and Aiken further showed that knockout of the IPMK gene resulted in a negligible effect on HIV-1 infection of T cells [7]. Because IP6 is incorporated into HIV-1 particles during assembly, it remains possible that virion-associated IP6 is sufficient for capsid stabilization during infection. Supporting this hypothesis, a CA mutant that assembles efficiently but does not encapsidate IP6 was found to contain defective cores, consistent with IP6 playing a critical role in capsid assembly and/or stability [56]. Other proteins or small molecules may also stabilize the capsid following HIV-1 entry into cells. While the currently available evidence suggests that virion IP6 plays roles in both assembly and maturation, understanding the role of the metabolite in early post-entry steps of infection awaits further studies.

The observation that capsid stabilization by IP6 permits efficient reverse transcription seems likely to lead to a greater understanding of HIV-1 reverse transcription in the natural context of the viral core. In the Christensen study, the ERT reaction was associated with partial capsid disassembly in vitro, providing an initial structural glimpse into the process of HIV-1 uncoating which is intimately connected to nuclear entry of the virus. Indeed, a recent imaging study shows that HIV-1 cores appear to enter the nucleus by penetrating nuclear pores in an intact or nearly intact state, after which they complete reverse transcription and undergo uncoating and integration [57]. These findings helped to validate an earlier study reporting the interaction of apparently intact HIV-1 cores with the nuclear envelope [58]. Following nuclear entry, binding of the nuclear host protein CPSF6 to the capsid is thought to stabilize it and promote penetration deep within the nucleus thus facilitating integration within gene-dense regions of chromatin [59]. HIV-1 uncoating is perturbed by an emerging class of antiviral compounds known as capsid inhibitors, and elucidating this viral process will be important for a complete understanding of their antiviral mechanisms [60].

As stated previously, early studies of endogenous reverse transcription in avian retroviruses indicated that these reactions could occur relatively efficiently in the absence of added host factors, raising the question as to how these reactions occur in the absence of capsid stabilization. Because the HIV-1 capsid has been studied more intensively than capsids of other retroviruses, the explanation for these apparent differences is not yet known. Capsid metastability may be a specific feature of lentiviruses, and it is possible that non-lentiviral capsids are inherently more stable. It is currently unknown whether the mature capsids of retroviruses of genera other than lentiviruses are also stabilized by cellular metabolites.

## Reverse transcription promotes HIV-1 uncoating

Using approaches involving immunofluorescent imaging and escape from host restriction, the Hope lab has shown that reverse transcription drives HIV-1 capsid disassembly (uncoating) from the core in target cells [61]. Consistent with this observation, we (Rousso’s team) employed atomic force microscopy (AFM) to study the mechanical properties of HIV-1 cores. In AFM, a cantilever probe is used to quantify the stiffness (resistance to deformation) as well as the shape of an object. AFM can be applied to biological structures under native conditions and is nondestructive, thus permitting analysis of changes in the properties of an object over time. In initial studies, we observed that native HIV-1 cores exhibit a greater stiffness than structures assembled in vitro from recombinant CA, suggesting that the core is a semi-solid object and that its physical properties depend on components in addition to the capsid [62, 63]. Capsid-stabilizing mutations increased the stiffness of HIV-1 cores, as did addition of the capsid-binding host protein cyclophilin A and the antiviral compound PF74 [63, 64]. These studies provided the first insights into the mechanical properties of the HIV-1 capsid that are likely to influence uncoating.

In subsequent studies, we employed AFM to analyze the effects of ERT on the properties of HIV-1 cores. Reverse transcription induced an increase in core stiffness. High resolution mechanical mapping of the core surface revealed the appearance of a striated pattern suggestive of a coiled structure beneath the capsid surface. Subsequently, the coiled structure vanished and the stiffness of the capsid dropped to a level below its initial value. Extended incubation for several hours resulted in a softening of the core and its eventual disassembly [65]. These results were consistent with reverse transcription-dependent uncoating in target cells described earlier [61].

Reverse transcription of retroviruses occurs in a series of stages (see [31] for a detailed review). Minus strand synthesis is initiated by extension of a host cell tRNA primer that is encapsidated during virion assembly. Extension from this primer by RT results in a 181-nucleotide DNA molecule that is complementary to the 5′-end of the viral genome. This molecule, termed the minus strand strong stop DNA, is released following degradation of the base-paired region of the genome by the ribonuclease H activity of RT, allowing its annealing to the repeat (R) sequence at the 3′-end of the genome in a reaction termed the minus strand transfer. Subsequent extension of the molecule by RT results in the full-length minus strand and degradation of the bulk of the RNA template by ribonuclease H digestion. Synthesis of the plus strand is primed by one or more RNA oligonucleotide remnants of the genome left behind by ribonuclease H degradation, including the polypurine tract. Extension of the PPT yields the plus strand strong stop DNA molecule, which subsequently anneals to a sequence in the 3′ end of the minus strand DNA in a reaction termed the plus strand transfer. Reverse transcription is completed when both strands are extended to generate the long terminal repeat (LTR) sequences at the ends of the double strand DNA product.

**Fig. 2:**
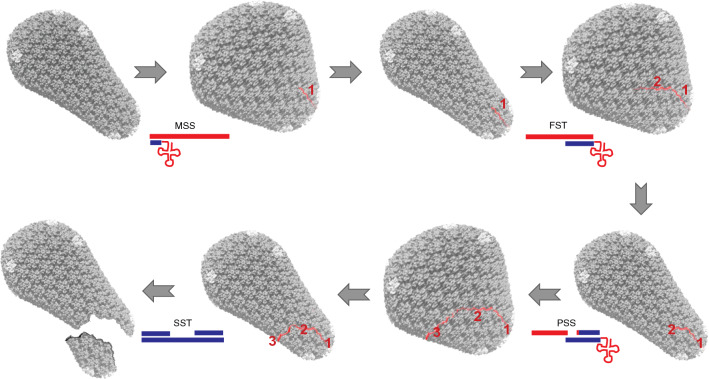
Illustration of a cumulative damage model for uncoating. During reverse transcription, the core undergoes a series of three stiffness spikes during which the core structure becomes transiently bloated. Each spike corresponds to a distinct reverse-transcription stage which generates a localized defect in the capsid structure. The first, second and third spikes result from: (1) synthesis of minus-strand strong stop DNA; (2) elongation of the minus strand following the first strand transfer; and (3) synthesis of plus strand strong stop DNA. As reverse transcription is completed, the accumulated damage weakens the capsid resulting in its breakage

Recently we analyzed the effects of IP6 on the dynamics of HIV-1 core stiffness changes during ERT [66]. Addition of IP6 to purified cores increased their stiffness even in the absence of reverse transcription, consistent with its capsid stabilizing effect. In ERT reactions containing IP6, we observed a series of three discrete stiffness spikes which corresponded temporally to specific stages of DNA synthesis—i.e., MSS synthesis, extension following minus strand transfer, and plus strand initiation. Each of the stiffness events was followed by a relaxation phase, with further incubation resulting in capsid disassembly (uncoating). Inhibition of reverse transcription during or shortly after the stiffness spikes prevented the observed uncoating effect, suggesting that the structural changes within the core occurring during a late stage of ERT eventually trigger uncoating. Based on these observations, we propose a model in which the viral capsid undergoes subtle but cumulative damage during reverse transcription that primes the core for uncoating (Fig. 2). Consistent with recently reported evidence that reverse transcription and uncoating are completed following entry of the HIV-1 core into the target cell nucleus [57, 67, 68], the uncoating trigger may involve a cellular activity, or it could relate to the mechanically induced uncoating we observed in ERT reactions. The intrinsic stability of the capsid may also need to be low enough to permit reverse transcription-induced uncoating, as the hyperstable E45A mutant capsid fails to undergo uncoating during ERT and exhibits impaired integration at moderate concentrations of IP6 [65]. Of note, reverse transcription-induced uncoating observed in vitro is consistent with the elegant cryoelectron tomography studies of Christensen et al*.* showing capsid opening and DNA emergence at a late stage of ERT [54].

While the molecular basis for reverse transcription-induced uncoating is unknown, it seems plausible that the structural changes in the ribonucleoprotein complex within the capsid that must occur during reverse transcription result in direct forces on the viral capsid, potentially damaging the lattice. Such stress may result from changes in the structural state of the nucleic acid. Double-strand DNA is more rigid than RNA which adopts a relatively compact tertiary structure based on flexibility in base pairing. Stress on the capsid could also result from changes in internal pressure of the core owing to osmotic effects. During DNA synthesis, addition of a nucleotide releases pyrophosphate and incorporation of a nucleotide into the DNA chain, resulting in a net charge increase of − 1. Hydrolysis of pyrophosphate to phosphate would further increase the net negative charge in the core by − 2. This could lead to an influx of water and ions, transiently increasing the pressure within the capsid. While this model is speculative, it is consistent with the kinetics of stiffness spike formation during ERT and seems to account for the rapid reversal of capsid stiffness to baseline with each spike. Nonetheless, it does not explain why capsid stiffness does not remain elevated during the prolonged stage of full-length minus strand synthesis.

The mechanical properties of the viral capsid are fascinating. The structure is variably curved and flexible, and it undergoes elastic deformation during reverse transcription. Moreover, addition of IP6 and the CA-targeting inhibitor PF74 stiffens the capsid. Using AFM operating in the ultrafast scanning mode, we have observed that capsid stiffening is associated with rounding of the core, which returns to an elongated shape upon relaxation [66]. This suggests that the flexibility of the capsid endows it with elastic properties which allow it to accommodate the stresses during reverse transcription without breaking. Nonetheless, completion of reverse transcription in vitro results in capsid failure, suggesting that the capsid may experience subtle damage during reverse transcription that primes it for uncoating following nuclear entry. The elastic property of the capsid may also help the core pass through the nuclear pore, whose diameter has been recently observed to be just large enough to accommodate the wide end of an intact HIV-1 core [57].

## Conclusions

Recent studies involving ERT reactions have established the essential role of the capsid in HIV-1 reverse transcription, but the mechanisms by which the capsid performs its critical functions remain to be determined. Biophysical studies have also revealed punctuated effects on the mechanical properties of the core during reverse transcription in vitro. As a system to study reverse transcription within the viral capsid and the structural consequences thereof, studies utilizing the ERT reaction are likely to provide further interesting insights into the key processes of HIV-1 reverse transcription, uncoating, nuclear import, and integration.

## Data Availability

N/A.

## Abbreviations

HIV-1: Human immunodeficiency virus type 1
ERT: Endogenous reverse transcription
IP5: Inositol pentakisphosphate
IP6: Inositol hexakisphosphate
IPMK: Inositol polyphosphate multikinase
IPPK: Inositol pentakisphosphate 2-kinase
RT: Reverse transcriptase protein
NC: Nucleocapsid protein
IN: Integrase protein
CA: Capsid protein
SP1: Spacer peptide 1
TRIM5: Tripartite motif 5 protein
eEF1A: Elongation factor 1A
eEF1G: Elongation factor 1G
